# Mercurius Vivus

**Published:** 1873-01

**Authors:** H. L. Amble


### Mercurius Vivus.

BY H. L. AMBLE, D. D. S., M. D.

Mercurius is one of those heroic drugs which by the discovery of homeopathy has been converted into a life-saving and health-restoring instrument of the healing art; aiding us in proclaiming that every true drug in the three kingdoms of nature symbolizes or represents some corresponding disease of the animal system. Like many other drugs, in its crude form it is too energetic in its action; many times by the use of large doses endangering the health and life of the patient. But let us develop its finer powers and effects by combination and trituration with a vehicle which will not change the practical facts in relation to its medicinal effects: thus it is made agreeable to take, and its action is controlled to such an extent that patients will be free from all liability to salivation or any of its concomitant evils.

The name hydrargyrum is derived from two Greek words: *udor* (water) and *arguros* (silver.) It was called mercury in honor of *Mercurius*, the messenger of the gods, whose fleetness and volatile character mercury was supposed to symbolize. When found pure in a metallic state it is in globules in the cavities of ores, or combined with silver. At the present time the mines of California are said to be the richest in the world.

Aristotle informs us that Dæclatus, who lived 1,300 years before Christ, communicated a power of motion to a wooden Venus, by pouring quicksilver into it. Discorides and Plinius believed that mercury destroyed the inner tissues by its weight. It was first employed for the cure of cutaneous affections, and acts principally upon mucous and serous membrane, nervous tissue and the osseous system. The purest quality is obtained by the distillation of artificial cinnabar, (six parts mercury and one of sulphur with iron fillings;) sulphuret of iron is thus formed, the mercury passing into the receiver which should be filled with water. The mercury is then collected on a piece of leather, and by means of a press all humidity is removed, leaving it a brilliant tin white.

To prepare this drug in the homeopathic manner we take one part of it pure, and triturate with nine parts sugar of milk; this is called the first trituration according to the decimal scale. The next trituration would be made by using one part of the first with nine parts more sugar of milk, thus producing the second trituration; and in this way carrying the potency high as you may desire. In this remedy, however, the potency after the third or fifth, is generally carried higher by using pure alcohol for dilution and dynamiza tion.

In effect on examining the degree of intensity with which the various attenuations act, we find that the diminution of their energy is in no way proportioned to the diminution of their matter. On the contrary we are aware that many substances, such as lycopodium and carbo-vegatabilis, in a state of concentration, have little or no action on the system, but often become powerful agents when we bring them to the second or third potency.

The specific peculiarity of the action of this remedy is in specific rapport with the character of dental neuralgia and pericementitis. This remedy is an individual, and as such has an individual sphere of action, determining specific changes in organic tissues, and holding particular relation to these tissues in disease. The disease is driven out of the tissue by a species of elective affinity, which exists between the drug force and the morbific principal, the result being a natural absorption or equilibration; this state making itself apparent by a gradual diminution of morbid phenomena and return to health. We know that the lymphatics arise by a net work of delicate structures, and are distributed throughout the whole animal economy, bearing a close resemblance to the venous system. It is in these lymphatics that the chyle and lymph circulate, being gradually conducted to their reservoirs, the right and left thoracic ducts, which empty into the right and left subclavian veins; this circulation evidently being carried on independent of the heart's action, by the contractibility of the coats of the lymphatics, whose functions are preliminary to those of the veins.

After coming thus far we will be able to understand something of the physiological action and therapeutic use of mercurius vivus, which will be at the same time both logical and consistent. Then we say it is the lymphatic system upon and in which this drug first makes its special point of attack in the living organism; and in these lymphatics it meets the unfriendly morbid force which it is called upon to subdue, by diminishing and paralyzing the action of the irritable lymphatic capillaries. These phenomena are attended with symptoms of congestion and vascular excitement, similar but not like true congestion of the sanguineous capillaries. Ever true to their instinct of absorption the lymphatics take up this remedy; thus through them and the venous system secondarily it is carried to the immediate seat of disease, where it hushes the enemy, restoring a normal condition.

Mercurius should be kept in a clean, dry, dark place, free from all odors; and when obtained should be of known purity, well and thoroughly prepared for use by trituration. It is well to abstain from all food and drink, as well as excessive bodily or mental exercise for half an hour after taking. Thus we do away with leeches, blisters, and their accompanying satellites, which are so digusting and disagreeable, often destroying the very thing they seek to save, and tormenting you with well meant cruelties.

